# Reference values for umbilical cord arterial pH by mode of birth in preterm, term and postterm infants: a population-based cohort study

**DOI:** 10.1186/s12884-026-08804-z

**Published:** 2026-02-15

**Authors:** Noor Abdulahad, Tiia-Marie Sundberg, Mehreen Zaigham

**Affiliations:** 1 Obstetrics and Gynecology, Institution of Clinical Sciences Lund, Lund University, Lund, Sweden; 2 Department of Obstetrics and Gynecology, Ystad Hospital, Ystad, Sweden; 3 Department of Obstetrics and Gynecology, Skåne University Hospital, Malmö, Sweden

**Keywords:** Cesarean birth, Instrumental birth, Gestational age, Mode of birth, Reference values, Umbilical cord pH, Vacuum extraction birth, Vaginal birth

## Abstract

**Background:**

Umbilical cord arterial pH (UApH) is the most objective measurement of the metabolic condition of the infant at birth. However, studies have identified changes in UApH with advancing gestational age, as there is limited data examining UApH changes with different modes of birth. The objective was to establish reference values for UApH according to mode of birth and to investigate the influence of gestational age on changes in UApH across different modes of birth.

**Methods:**

A retrospective cohort study using data from Perinatal Revision South register, from seven maternity units in southern Sweden. Validated pH values from 103,115 infants were included. Cohort characteristics were reported collectively and for each mode of birth using numbers and percentages. Reference values were reported as 2.5th to 97.5th percentiles, with mean and median values. One-way ANOVA was used to investigate differences in UApH across different modes of birth. Simple and multiple linear regressions were performed associating gestational age with pH by mode of birth.

**Results:**

Mode of birth in the study population consistent of vaginal non-instrumental (80,957/78.5%), vacuum-assisted (6,486/6.3%), forceps-assisted (429/0.4%), elective Cesarean sections (6,295/6.1%), emergency Cesarean sections (8,180/7.9%), and immediate Cesarean sections (768/0.7%). UApH median values for vaginal non-instrumental births, elective Cesarean sections, and emergency Cesarean sections were 7.240, 7.290, and 7.276 respectively. Lower median values were observed for immediate Cesarean sections (Median = 7.180), vacuum-assisted (Median = 7.170), and forceps-assisted (Median = 7.170) births. A significant negative linear relationship was identified between gestational age and UApH in vaginal non-instrumental, emergency Cesarean sections, and vacuum-assisted vaginal births (*P* < 0.001).

**Conclusion:**

Reference values were outlined for UApH based on the mode of birth and gestational age. A trend of advancing acidosis was found in infants with increasing gestational age in non-instrumental vaginal births, emergency Cesarean sections, and vacuum-assisted births. Immediate Cesarean sections and instrumental births exhibited higher levels of acidosis.

## Introduction

Timely interventions used appropriately in obstetric care can be crucial in preventing perinatal morbidity and mortality [1]. Obstetricians rely on skill and experience to achieve high-quality decision-making for interventions when medically indicated [2]. Nevertheless, distribution of modes of birth varies significantly between regions of the world, regardless of adjustments for maternal and perinatal characteristics [3].

The risk management and quality of obstetric care can be objectively measured by using umbilical cord blood pH [4]. These blood gases can be used to judge the metabolic condition of the infant at birth and can help in the early identification of infants suffering from birth asphyxia, a leading cause of neonatal mortality worldwide [5–7]. However, since the physiology of different modes of birth predisposes fetuses to varying degrees of hypoxia, from little to no hypoxia in planned Cesarean sections (CS) to high levels during vaginal birth, it is important for obstetricians to be aware of such differences when interpreting umbilical cord blood gas values [8, 9].

The most common medical conditions resulting in emergency CS are dystocia, and abnormal fetal heart rate monitoring [10]. Operative vaginal birth is mainly indicated during the second stage of labor due to dystocia and/or clinical signs suggesting fetal acidemia [11]. Use of forceps have declined in the last decades, and compared to vacuum extraction (VE) is correlated to higher incidence of obstetric sphincter injuries [11]. Apart from Apgar score, clinical assessment of the infant is made through analyzing various blood gas parameters of the umbilical cord blood samples, reflecting the acid-base status of the infant [5, 12, 13]. Umbilical cord arterial pH (UApH) has been shown to reflect the metabolic state of the infant during birth, associated strongly with short-term morbidity and mortality in infants suffering from birth asphyxia [14, 15]. The definition of pathological UApH in infants varies among different studies, ranging between < 7.00 to < 7.20 [16, 17].

Previous research has however found that umbilical cord blood pH is not similar across different modes of birth [8, 18]. Yoon et al. observed significant differences in umbilical cord pH between vaginal births and CS in a cohort of low-risk singleton term pregnancies, with a decline in pH noted in vaginal births [18]. However, a study evaluating reference values for umbilical cord blood gases in spontaneous vaginal births and CS found no significant differences in UApH between the two modes of birth [19]. Complicating the interpretation of umbilical cord pH levels further, variations have been identified with respect to GA, where an inverse association has been established [9, 8, 20]. Notably, these studies included only normal, vaginal births and infants presenting with high Apgar scores (≥ 7) [8, 20]. One study observed that changes in mean arterial pH values ranged from 7.29 ± 0.10 at 28–31 weeks of gestation to 7.23 ± 0.07 at 42 completed weeks of gestation [20]. A normal range for a vaginal birth in a term infant may therefore not be applicable to infants born through other modes of birth at different GA.

Our aim was to investigate changes in UApH across modes of birth in preterm, term, and postterm infants, in order to characterize mode-of-birth-specific UApH values. Further, we aimed to examine the association between GA and UApH within each mode of birth.

## Methods

### Study design and setting

We conducted a retrospective, population-based cohort study in the south of Sweden using data extracted from the Perinatal Revision South (PRS) register, a regional register containing obstetric and neonatal data from seven Maternity Units in southern Sweden covering the years from 1995 to 2015.

### Study population

**Fig. 1:**
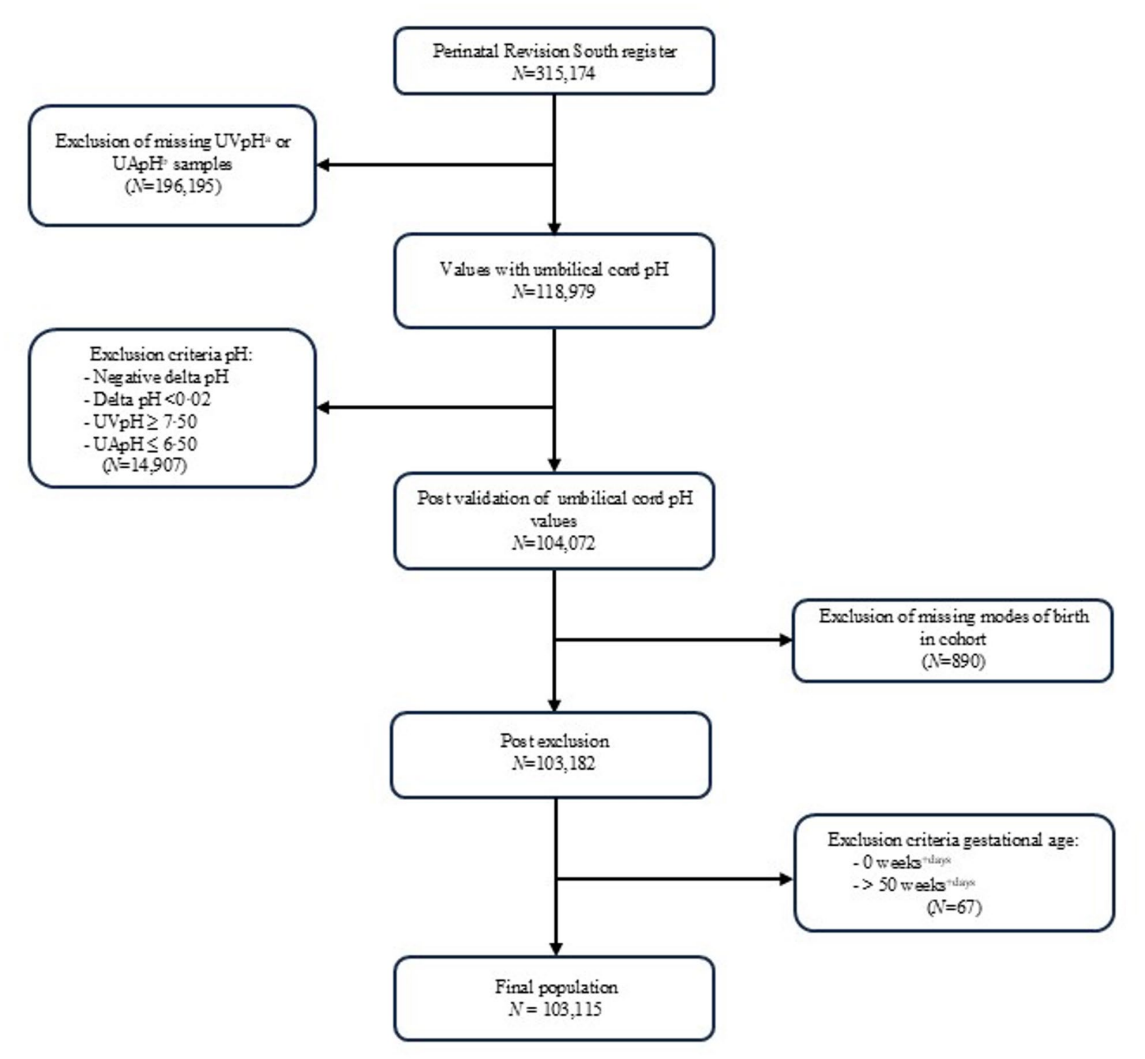
Flow diagram over selection of study population through validation of pH values and exclusion of aberrant or missing data

Obstetric and medical data from approximately 315,174 live births are recorded in the PRS database. The study included singleton births, including all GA, ranging from preterm (≤ 36 + 6) to term (37 + 0–41 + 1), and postterm (≥ 42 + 0) births. After data extraction from the database, initial exclusion criteria included multiple births and intrauterine fetal death. Thereafter, to ensure two-vessel sampling, all cases with missing cord arterial or venous pH were excluded. Blood gas values were rigorously validated using calculations of venoarterial differences (delta pH) between umbilical cord venous and arterial pH, excluding samples with delta pH < 0.02, assuring correct blood sampling [21]. Venous pH values of ≥ 7.50 and arterial values of ≤ 6.50 were considered erroneous and excluded. As the study was conducted according to mode of birth, cases with unreported mode of birth were eliminated. Finally, GA reported as 0 days or ≥ 50 weeks were considered unphysiological and excluded, resulting in a final study population of 103,115 cases. Exclusion criteria and cohort selection are presented in Fig. 1.

### Statistical analysis

Reference values for UApH were computed and presented based on mode of birth and GA (in weeks), with all preterm births collected into one group (≤ 36 + 6). Mode of birth were divided into vaginal non-instrumental, vaginal forceps, vaginal VE, elective CS, emergency CS and immediate CS. Subsequently, frequency tables were generated empirically, encompassing mean values, as well as the 2.5th, 5th, 50th, 95th, and 97.5th percentiles for UApH. Using total mean values across each mode of birth, one-way analysis of variance (ANOVA) was conducted to determine if differences in UApH existed for groups undergoing different modes of birth.

The association between GA and UApH was studied with simple linear regression. Associations were investigated according to each mode of birth separately, allowing for independent assessments. Thereafter, a multiple linear regression model was constructed, adjusting for birth weight as a second independent variable, generating adjusted calculations for changes in GA with UApH. A *p*-value of < 0.05 was considered statistically significant. Before statistical analysis, assumptions for each analysis were checked. All data analysis was performed using IBM SPSS Statistics 27.

## Results

Baseline characteristics of the study population are outlined in Table 1. The majority of the study population consisted of non-instrumental vaginal birth, while immediate CS (768/103,115, 0.7%) and forceps-assisted births were more rarely observed. Details of baseline characteristics according to mode of birth are outlined in Table 2. Differences regarding parity were observed when considering the mode of birth. Most women that had a vaginal non-instrumental birth (45,351/80,957, 56.0%) and elective CS (4,185/6,295, 66.5%) were multiparous. Higher percentages of women in groups of all forms of CS were found to had undergone previous CS. A higher frequency of lower Apgar scores was noted in infants born through immediate CS, emergency CS and instrumental vaginal births. Missing values for BMI were reported in 41,558 cases, constituting 40% of the cohort.

**Table 1:** Demographic characteristics of total study population

Total	*N*	%
	103,115	100
Maternal Age (years)		
< 20	1,966	1.9
20–34	81,128	78.7
35–39	16,761	16.3
≥ 40	3,260	3.2
Parity		
Primipara	48,586	47.1
Multipara	54,529	52.9
Previous CS^a^		
No	97,779	94.8
Yes	5,336	5.2
Maternal BMI^b^ (kg/m^2^)		
< 18.5	1,417	1.4
18.5–24.9	35,409	34.3
25–29.9.9	16,372	15.9
≥ 30	8,359	8.1
Missing	41,558	40.3
Maternal Smoking (cigarettes/day)		
No	97,044	94.1
Yes (1–9)	4,424	4.3
Yes (≥ 10)	1,647	1.6
Birth Start		
Spontaneous	85,952	83.4
Induction	9,849	9.6
CS	7,304	7.1
Missing	10	0.0
Mode of Birth		
Vaginal, non-instrumental	80,957	78.5
CS, elective	6,295	6.1
CS, emergency	8,180	7.9
CS, immediate	768	0.7
Vaginal, VE^c^	6,486	6.3
Vaginal, forceps	429	0.4
Gestational Age (weeks^+days^)		
Extremely and very preterm (< 32^+ 0^)	442	0.4
Preterm (32^+ 0^−36^+ 6^)	4,787	4.6
Term (37^+ 0^−40^+ 6^)	76,187	73.9
Late term (41^+ 0^−41^+ 6^)	16,165	15.7
Postterm (≥ 42^+ 0^)	5,534	5.4
Sex		
Female	49,395	47.9
Male	53,640	52.0
Unknown	80	0.1
Birth Weight (grams)		
< 2500	2,792	2.7
2500–3999	80,074	77.7
4000–4499	16,063	15.6
≥ 4500	4,123	4.0
Missing	63	0.1
5-minute Apgar Score		
0–3	206	0.2
4–6	878	0.9
7–10	102,011	98.9
Missing	20	0.0

^a^*CS *Cesarean section, ^b^*BMI *Body Mass Index, ^c^*VE*: Vacuum Extraction

**Table 2:** Characteristics of study population per mode of birth

	Vaginal, non instrumental		CS^a^ elective		CS emergency		CS immediate		Vaginal VE^b^		Vaginal Forceps	
	*N*	%	*N*	%	*N*	%	*N*	%	*N*	%	*N*	%
Total	80,957	100	6,295	100	8,180	100	768	100	6,486	100	429	100
Maternal age (years)												
< 20	1,696	2.1	40	0.6	102	1.2	11	1.4	114	1.8	3	0.7
20–34	64,726	80.0	4,140	65.8	6,097	74.5	574	74.7	5,249	80.9	342	79.7
35–39	12,370	15.3	1,656	26.3	1,579	19.3	135	17.6	950	14.6	71	16.6
≥ 40	2,165	2.7	459	7.3	402	4.9	48	6.3	173	2.7	13	3.0
Parity												
Primipara	35,606	44.0	2,110	33.5	5,071	62.0	414	53.9	5,045	77.8	340	79.3
Multipara	45,351	56.0	4,185	66.5	3,109	38.0	354	46.1	1,441	22.2	89	20.7
CS previously												
No	78,479	96.9	4,809	76.4	7,279	89.0	683	88.9	6,124	94.4	405	94.4
Yes	2,478	3.1	1,486	23.6	901	11.0	85	11.1	362	5.6	24	5.6
Maternal BMI^c^ (kg/m^2^)												
< 18.5	1,151	1.4	63	1.0	82	1.0	11	1.4	100	1.5	10	2.3
18.5–24.9	27,957	34.5	1,929	30.6	2,376	29.0	289	37.6	2,682	41.4	176	41.0
25.0–29.9.0.9	12,315	15.2	1,141	18.1	1,519	18.6	167	21.7	1,164	17.9	66	15.4
≥ 30.0	5,991	7.4	705	11.2	1,042	12.7	120	15.6	468	7.2	33	7.7
Missing	33,543	41.4	2,457	39.9	3,161	38.6	181	23.6	2,072	31.9	144	33.6
Maternal smoking (cigarettes/day)												
No	76,168	94.1	5,920	94.0	7,719	94.4	688	89.6	6,148	94.8	401	93.5
Yes (1–9)	3,467	4.3	264	4.2	346	4.2	61	7.9	266	4.1	20	4.7
Yes (≥ 10)	1,322	1.6	111	1.8	115	1.4	19	2.5	72	1.1	8	1.9
Birth start												
Spontaneous	73,831	91.2	89	1.4	5,525	67.5	546	71.1	5,593	86.2	368	85.8
Induction	7,108	8.8	14	0.2	1,624	19.9	149	19.4	893	13.8	61	14.2
CS	11	0.0	6,192	98.4	1,029	12.6	72	9.4	0	0.0	0	0.0
Missing	7	0.0	0	0.0	2	0.0	1	0.1	0	0.0	0	0.0
Gestational age (weeks^+days)^												
Extremely and very preterm (< 32^+ 0^)	196	0.2	12	0.2	217	2.7	15	2.0	1	0.0	1	0.2
Preterm (32^+ 0^−36^+ 6^)	3,197	3.9	231	3.7	1,076	13.2	73	9.5	190	2.9	20	4.7
Term (37^+ 0^−40^+ 6^)	60,954	75.3	5,893	93.6	4,447	54.4	411	53.5	4,199	64.7	283	66.0
Late term (41^+ 0^−41^+ 6^)	12,883	15.9	87	1.4	1,475	18.0	158	20.6	1,474	22.7	88	20.5
Postterm (≥ 42^+ 0^)	3,727	4.6	72	1.1	965	11.8	111	14.5	622	9.6	37	8.6
Sex assigned at birth												
Female	39,498	48.8	3,079	48.9	3,547	43.4	300	39.1	2,800	43.2	171	39.9
Male	41,397	51.1	3,212	51.0	4,627	56.6	468	60.9	3,678	56.7	258	60.1
Unknown	62	0.1	4	0.1	6	0.1	0	0	8	0.1	0	0.0
Birth weight (grams)												
< 2500	1,492	1.8	187	3.0	944	11.5	67	8.7	92	1.4	10	2.3
2500–3999	63,854	78.9	5,055	80.3	5,244	64.1	566	73.7	5,018	77.4	337	78.6
4000–4499	12,639	15.6	731	11.6	1,398	17.1	103	13.4	1,123	17.3	69	16.1
≥ 4500	2,937	3.6	315	5.0	586	7.2	28	3.6	246	3.8	11	2.6
Missing	35	0.0	7	0.1	8	0.1	4	0.5	7	0.1	2	0.5
5-minute Apgar Score												
0–3	83	0.1	7	0.1	51	0.6	43	5.6	18	0.3	4	0.9
4–6	354	0.4	37	0.6	234	2.9	69	9.0	168	2.6	16	3.7
7–10	80,507	99.4	6,251	99.3	7,889	96.4	656	85.4	6,299	97.1	409	95.3
Missing	13	0.0	0	0.0	6	0.1	0	0.0	1	0.0	0	0.0

^a^ *CS *Cesarean Section, ^b^*VE *Vacuum Extraction, ^c^*BMI *Body Mass Index

Tables 3, 4, 5, 6, 7 and 8 present UApH reference values according to mode of birth and GA in weeks. In vaginal non-instrumental births, a clear trend of decreasing UApH was found with advancing GA. The median UApH value for preterm births was 7.300 and for postterm births 7.220 (Table 3). In elective CS a similar trend was found but to a lesser extent, with a median arterial pH of 7.300 in preterm infants and 7.287 in late term gestations (Table 4). For emergency CS, median UApH for preterm gestations was 7.290 and 7.270 for postterm gestations (Table 5). Reference values for immediate CS were less consistent. However, a decline in median UApH with advancing GA was still found: 7.210 for preterm and 7.190 for postterm infants (Table 6). Consistent paradoxical trends between GA and UApH were found in the reference values for VE births, with a median UApH in preterm births was 7.190 compared to 7.160 in postterm births (Table 7). For forceps-assisted births, a decrease was found in UApH comparing preterm births (Median = 7.180) to late term gestations (Median = 7.145) (Table 8). However, for postterm gestations, median UApH was 7.170.

**Table 3:** Reference values for umbilical cord arterial pH for vaginal non-instrumental births relative to gestational age at birth

Gestational age (weeks^+days^)	*N*	2.5th	5th	Percentiles (UApH^a^) 50th (Median)	95th	97.5th	Mean (UApH)
Total	80,957	7.070	7.100	7.240	7.350	7.370	7.236
< 32^+ 0^–36^+ 6^	3,393	7.080	7.159	7.300	7.412	7.441	7.289
37^+ 0^–37^+ 6^	4,204	7.080	7.115	7.253	7.360	7.380	7.247
38^+ 0^–38^+ 6^	10,696	7.090	7.120	7.250	7.360	7.380	7.246
39^+ 0^–39^+ 6^	21,807	7.070	7.110	7.250	7.350	7.370	7.241
40^+ 0^–40^+ 6^	24,247	7.070	7.100	7.240	7.350	7.370	7.232
41^+ 0^–41^+ 6^	12,883	7.56	7.090	7.230	7.340	7.360	7.223
≥ 42^+ 0^	3,727	7.040	7.070	7.220	7.330	7.350	7.212

^a^*UApH *Umbilical cord arterial pH

**Table 4:** Reference values for umbilical cord arterial pH for elective Cesarean sections relative to gestational age at birth

Gestational age (weeks^+days^)	*N*	2.5th	5th	Percentiles (UApH^a^) 50th (Median)	95th	97.5th	Mean (UApH)
Total	6,295	7.110	7.160	7.290	7.350	7.360	7.279
< 32^+ 0^–36^+ 6^	243	7.131	7.182	7.300	7.350	7.370	7.287
37^+ 0^–37^+ 6^	652	7.103	7.170	7.290	7.340	7.350	7.277
38^+ 0^–38^+ 6^	3850	7.110	7.162	7.290	7.350	7.360	7.280
39^+ 0^–39^+ 6^	1209	7.100	7.160	7.290	7.350	7.360	7.279
40^+ 0^–40^+ 6^	182	7.079	7.120	7.290	7.342	7.350	7.273
41^+ 0^–41^+ 6^	87	7.062	7.111	7.287	7.356	7.376	7.269
≥ 42^+ 0^	72	7.185	7.200	7.291	7.344	7.359	7.289

^a^*UApH *Umbilical cord arterial pH

**Table 5:** Reference values for umbilical cord arterial pH for emergency Cesarean sections relative to gestational age at birth

Gestational age (weeks^+days^)	*N*	2.5th	5th	Percentiles (UApH^a^) 50th (Median)	95th	97.5th	Mean (UApH)
Total	8,180	7.050	7.110	7.276	7.350	7.360	7.260
< 32^+ 0^–36^+ 6^	1,293	7.030	7.100	7.290	7.351	7.361	7.270
37^+ 0^–37^+ 6^	647	7.062	7.130	7.290	7.350	7.360	7.272
38^+ 0^–38^+ 6^	961	7.070	7.130	7.280	7.350	7.360	7.265
39^+ 0^–39^+ 6^	1,191	7.038	7.090	7.270	7.340	7.360	7.253
40^+ 0^–40^+ 6^	1,648	7.060	7.107	7.270	7.340	7.350	7.253
41^+ 0^–41^+ 6^	1,475	7.059	7.110	7.270	7.340	7.350	7.250
≥ 42^+ 0^	965	7.030	7.110	7.270	7.340	7.350	7.252

^a^*UApH *Umbilical cord arterial pH

**Table 6:** Reference values for umbilical cord arterial pH for immediate Cesarean sections relative to gestational age at birth

Gestational age (weeks^+days^)	*N*	2.5th	5th	Percentiles (UApH^a^) 50th (Median)	95th	97.5th	Mean (UApH)
Total	768	6.823	6.895	7.180	7.310	7.330	7.155
< 32^+ 0^–36^+ 6^	88	6.689	6.730	7.210	7.326	7.350	7.158
37^+ 0^–37^+ 6^	36	6.790	6.833	7.145	7.332	-	7.135
38^+ 0^–38^+ 6^	69	6.860	6.876	7.170	7.305	7.328	7.150
39^+ 0^–39^+ 6^	137	6.874	6.920	7.190	7.320	7.338	7.158
40^+ 0^–40^+ 6^	169	6.783	6.890	7.180	7.300	7.340	7.154
41^+ 0^–41^+ 6^	158	6.833	6.900	7.170	7.310	7.320	7.149
≥ 42^+ 0^	111	6.876	7.010	7.190	7.300	7.332	7.170

Groups with too few cases did not yield any results

^a^*UApH *Umbilical cord arterial pH

**Table 7:** Reference values for umbilical cord arterial pH for instrumental vaginal births using vacuum extraction relative to gestational age at birth

Gestational age (weeks^+days^)	*N*	2.5th	5th	Percentiles (UApH^a^) 50th (Median)	95th	97.5th	Mean (UApH)
Total	6,486	6.990	7.020	7.170	7.290	7.319	7.166
< 32^+ 0^–36^+ 6^	191	7.028	7.050	7.190	7.310	7.330	7.190
37^+ 0^–37^+ 6^	268	6.990	7.032	7.190	7.329	7.350	7.183
38^+ 0^–38^+ 6^	567	6.990	7.020	7.180	7.290	7.320	7.172
39^+ 0^–39^+ 6^	1362	6.987	7.021	7.170	7.301	7.320	7.170
40^+ 0^–40^+ 6^	2002	6.990	7.020	7.170	7.290	7.310	7.164
41^+ 0^–41^+ 6^	1474	6.989	7.030	7.170	7.280	7.310	7.162
≥ 42^+ 0^	622	7.000	7.030	7.160	7.278	7.290	7.157

^a^*UApH *Umbilical cord arterial pH

**Table 8:** Reference values for umbilical cord arterial pH for instrumental vaginal births using forceps relative to gestational age at birth

Gestational age (weeks^+days^)	*N*	2.5th	5th	Percentiles (UApH^a^) 50th (Median)	95th	97.5th	Mean (UApH)
Total	429	6.968	7.000	7.170	7.300	7.320	7.160
<32^+ 0^–36^+ 6^	21	7.000	7.000	7.180	7.280	-	7.167
37^+ 0^–37^+ 6^	19	6.900	6.900	7.160	-	-	7.156
38^+ 0^–38^+ 6^	35	6.850	6.978	7.150	7.326	-	7.147
39^+ 0^–39^+ 6^	95	6.980	6.998	7.170	7.320	7.338	7.163
40^+ 0^–40^+ 6^	134	6.993	7.020	7.180	7.290	7.306	7.174
41^+ 0^–41^+ 6^	88	6.817	6.944	7.145	7.286	7.307	7.141
≥ 42^+ 0^	37	6.939	7.949	7.170	7.311	-	7.157

Groups with too few cases did not yield any results

^a^*UApH *Umbilical cord arterial pH

Comparable total UApH values were observed for vaginal non-instrumental births, elective as well as emergency CS. Median UApH for vaginal non-instrumental births was 7.240, 7.290 for elective CS, and 7.276 for emergency CS. Consequently, lower values of UApH were found in infants born by immediate CS (Median = 7.180), VE (Median = 7.170), and forceps-assisted births (Median = 7.170). One-way ANOVA showed significant differences in UApH across modes of birth disregarding GA (*P* < 0.001).

Simple linear regression investigating changes in UApH with increasing GA at birth showed a negative linear relationship across all modes of birth but one, immediate CS (Table 9). A significant decrease in UApH was observed with increasing GA in vaginal non-instrumental births, emergency CS and VE births. When adjusting for birth weight in the multiple regression analyses, significant negative associations remained. There was a small increase in *R*^*2*^ indicating that birth weight as a predictor accounted for a small proportion of the variance in UApH and GA accounted for the majority.

**Table 9:** Simple and multiple linear regression analysis showing influence of gestational age (days) on umbilical cord arterial pH in each mode of birth

		Unadjusted				Adjusted^a^			
	*N*	Coefficient	95% CI^b^	*P*-value	*R* ^2^	Coefficient	95% CI	*P*-value	*R* ^2^
Vaginal non-instrumental	80,957	− 0.0009	− 0.0010 to − 0.0009	< 0.001	0.016	− 0.0008	− 0.0008 to − 0.0007	< 0.001	0.017
CS^c^ elective	6,295	− 0.0002	− 0.0004 to 0.00004	0.109	0.000	0.00002	− 0.00001 to 0.0004	0.065	0.007
CS emergency	8,180	− 0.0004	− 0.0004 to − 0.0003	< 0.001	0.008	− 0.0005	− 0.0006 to − 0.0004	< 0.001	0.009
CS immediate	768	0.00001	− 0.0005 to 0.0005	0.983	0.000	0.0002	− 0.0006 to 0.0009	0.662	0.001
Vaginal VE^d^	6,486	− 0.0007	− 0.0009 to − 0.0005	< 0.001	0.008	− 0.0009	− 0.0011 to − 0.0007	< 0.001	0.009
Vaginal forceps	429	− 0.0003	− 0.0010 to 0.0004	0.409	0.002	− 0.0005	− 0.0014 to 0.0003	0.230	0.004

^a^Adjusted for birth weight, ^b^*CI *Confidence interval, ^c^*CS *Cesarean section, ^d^*VE *Vacuum extraction

## Discussion

In this large population-based study, we examined changes in UApH across modes of birth in preterm, term, and postterm gestations. Reference values for UApH were provided and UApH was found to decrease linearly with increasing GA in normal vaginal births, emergency CS, and VE births. As expected, infants undergoing immediate CS, VE, or forceps-assisted births demonstrated lower UApH values compared to those born by vaginal non-instrumental births, elective CS, or emergency CS. Previous research has shown that umbilical cord blood pH changes with advancing GA [8, 9, 20, 22, 23]. However, the study settings were limited to vaginal non-instrumental births, where several exclusion criteria were applied to ensure the inclusion of only vigorous infants [8, 9, 20, 22]. In contrast, this study included infants in all clinical states, intending to represent the distribution of a general population. Currently, a literature gap exists regarding reference values for UApH according to the mode of birth of the infant and the mechanisms in which GA impacts umbilical cord pH. This study revealed comparable median values for ‘normal’ vaginal births, elective and emergency CS. Lower values were observed for immediate CS, VE, and forceps-assisted births. Vaginal births, including both non-instrumental and instrumental, have been shown to have lower UApH as compared to elective CS compared to emergency CS [8, 18, 23]. Kotaska et al. re-evaluated reference ranges for UApH and found no significant differences in values in infants born by non-instrumental vaginal birth compared with CS.^19^ Nickelsen et al. demonstrated more acidotic values in infants born by instrumental vaginal births and higher UApH values in CS than in spontaneously born infants [24]. A suggested explanation for the higher UApH following CS is that all CS were performed before the start of the active phase of labor, excluding the mother actively pushing, thereby decreasing the anaerobic effort in birth on the infant, since acidemia has been found to increase with the length of the second stage of labor [9, 24]. A study conducted by Prior et al. showed that modes of birth independently influence the condition of the infant at birth, with worst outcomes observed in instrumental vaginal births due to fetal compromise interpreted through a cardiotocograph compared to vaginal non-instrumental birth and emergency CS.^25^ Instrumental vaginal births have previously been associated with worse outcomes in infants at birth [25]. Furthermore, as previously mentioned, UApH values below 7.00 have been strongly associated with worse neonatal outcomes, with an increased risk showing at 7.05 [14, 26]. In accordance with the literature, we found that infants born by instrumental vaginal births were more acidotic, with a greater proportion of acidemia < 7.00 at higher percentiles, a clinically relevant association. Moreover, higher incidences of low and moderately abnormal Apgar scores were found in these groups compared to the total population. Therefore, lower ranges of UApH in infants born through instrumental births or immediate CS are presumed to reflect infants with compromised conditions in our cohort. To investigate this further, associations with neonatal outcomes should be examined.

### Clinical relevance

There is no consensus regarding the appropriate UApH cutoff value for what is considered fetal acidosis at birth. Different definitions exist in the literature and range most commonly between values of 7.00 to 7.20 [14, 16, 17, 26–28]. The use of stationary values clinically for infants of all GA has been questioned previously, and our findings of differences across modes of births and GA-related changes in UApH align with this reasoning [22]. The decline in UApH with advancing pregnancy means that higher UApH values are expected in premature infants, implying risk of underdiagnosing birth asphyxia in this preterm population. However, clinical indications for interventions, i.e., modes of birth deviating from vaginal non-instrumental births, are not reported and therefore this study presents correlation of UApH to mode of birth, not causal relationships. As expected, effect sizes were relatively small, with UApH decreasing with − 0.0008 (95%CI − 0.0008 - − 0.0007) per day for vaginal non-instrumental birth compared to emergency CS − 0.0005 (95%CI − 0.0006 - − 0.0004), suggesting limited clinical significance. To our knowledge, there is no literature studying the combined changes of UApH according to both GA and mode of birth. The establishment of reference values has significant clinical importance as they will help facilitate the correct evaluation of UApH in infants and contribute to enhanced obstetric management. Our study takes steps toward bringing clarity to the controversy of what is deemed a ‘normal’ and ‘abnormal’ UApH at birth. However, this study has not compared UApH to neonatal outcomes, which is relevant for determining pathological cut-offs. Therefore, further research is required to investigate the effect mode of birth and GA have on UApH and correlate it to neonatal outcomes.

### Strengths and limitations

The main strength of this study lies in the use of a large and robust cohort, which accurately represents the general population. Swedish healthcare registries are commonly acknowledged for holding high quality [29]. When similar studies have been conducted in the past, strict exclusion criteria were applied ensuring a healthy population and resulting in too few cases of extreme prematurity. In this study, our approach aimed to represent an actual population by including infants of all clinical states. The inclusion of all modes of birth and infants of all Apgar scores resulted in a sufficiently large cohort allowing the inclusion of extremely premature infants. However, since the cohort reflects the population of mothers and infants in the south of Sweden from 1995 to 2015, there is an inherent uneven distribution of groups according to mode of birth, leading to less reliable results in the groups with fewer numbers. As covariate, BMI was previously not reported for all births to the database, with 40% missing values in our study population. This may introduce bias if missingness was not completely at random and should be considered when interpreting the results. Cord blood sampling has been a routine procedure in Skåne since 1981. Strict validation of all umbilical cord pH values adds strength by excluding potentially faulty values that otherwise may have skewed the results. In this study, birth weight was considered and adjusted for the statistics as a potential confounder. Another limitation is that effect modifiers were not considered in this study.

## Conclusions

This study evaluated UApH across the different modes of birth commonly seen in the clinical setting and stratified changes in UApH according to GA at birth in a population of 103,115 infants. UApH was found to decrease linearly with GA in vaginal non-instrumental births, emergency CS, and VE births. Infants born by immediate CS, VE or forceps-assisted births were found to be more acidotic. Detailed reference values were provided for different GA across each mode of birth which can be used in clinical practice. Further research investigating the influence mode of birth has on UApH and associating the current findings with neonatal outcomes is required to gain more understanding and to clinical management of birth.

## Data Availability

The data and materials are according to the Ethical approval from the Swedish Ethical Review Authority not publicly available, but the authors can upon request provide data and materials.

## Abbreviations

BMI: Body mass index
CS: Cesarean section
GA: Gestational age
UApH: Umbilical cord arterial pH
VE: Vacuum extraction
